# Exploring Implementation of Personal Breast Cancer Risk Assessments

**DOI:** 10.3390/jpm11100992

**Published:** 2021-09-30

**Authors:** Maria A. Sierra, Jack C. W. Wheeler, Lisa Devereux, Alison H. Trainer, Louise Keogh

**Affiliations:** 1 Miller School of Medicine, University of Miami, Miami, FL 33136, USA; mariasierra@med.miami.edu; 2 Centre for Health Equity, Melbourne School of Population and Global Health, The University of Melbourne, Melbourne, VIC 3000, Australia; Jack.Wheeler@petermac.org (J.C.W.W.); l.keogh@unimelb.edu.au (L.K.); 3 Parkville Familial Cancer Centre, Melbourne, VIC 3000, Australia; 4 Peter MacCallum Cancer Centre, Melbourne, VIC 3000, Australia; 5 Research Division, Sir Peter MacCallum Department of Oncology, The University of Melbourne, Parkville, VIC 3000, Australia; lisa.devereux@petermac.org; 6 Department of Medicine, The University of Melbourne, Melbourne, VIC 3000, Australia

**Keywords:** risk-stratified breast screening, polygenic risk, implementation, breast cancer risk

## Abstract

Personal Breast Cancer (BC) Risk Assessments (PBCRA) have potential to stratify women into clinically-actionable BC risk categories. As this could involve population-wide genomic testing, women’s attitudes to PBCRA and views on acceptable implementation platforms must be considered to ensure optimal population participation. We explored these issues with 31 women with different BC risk profiles through semi-structured focus group discussions or interviews. Inductive thematic coding of transcripts was performed. Subsequently, women listed factors that would impact on their decision to participate. Participants’ attitudes to PBCRA were positive. Identified themes included that PBCRA acceptance hinges on result actionability. Women value the ability to inform decision-making. Participants reported anxiety, stress, and genetic discrimination as potential barriers. The age at which PBCRA was offered, ease of access, and how results are returned held importance. Most women value the opportunity for PBCRA to inform increased surveillance, while highlighting hesitance to accept reduced surveillance as they find reassurance in regular screening. Women with *BRCA* pathogenic variants value the potential for PBCRA to identify a lower cancer risk and potentially inform delayed prophylactic surgery. This study highlights complexities in adopting advances in BC early detection, especially for current users who value existing processes as a social good.

## 1. Introduction

Breast cancer (BC) is a heterogeneous disease, comprising cancers with markedly different prognostic and predictive characteristics [1]. It is the most common cancer in women and one of the leading causes of death worldwide, impacting approximately 2.3 million women in 2020 [2].

Although the incidence of BC continues to increase, breast cancer mortality is improving due to improved treatment regimes, early-stage detection through national population breast cancer screening programs (NBSP), and more targeted risk-mitigation strategies in those women at the highest risk [3,4].

Recent advances in our ability to quantify and model BC risk factors provide the potential for the introduction of more personalized BC risk assessments, and the adoption of a risk-stratified screening approach across the population [5].

Genome-wide association studies have identified over 300 common single nucleotide polymorphisms (SNPs) associated with breast cancer risk [5,6,7]. Individually, the risk attributable to each low-penetrance allele is small. However, the aggregate lifetime BC risk conferred by multiple variants, denoted as a polygenic risk score (PRS), can exceed 30% [5]. PRS, in addition to other lifestyle and physiological risk factors, can be used to assign personalized BC risk assessments (PBCRA) using multifactorial clinical models such as CANRISK irrespective of family history of the disease [8,9]. This approach also provides personal modified risk assessments to women with pathogenic variants in moderate or high-risk genes identified through family-based genetic testing in familial cancer clinics (FCC), or through nascent *BRCA* population screening studies [10,11].

Although issues relating to methodology, personal and clinical utility are still being assessed, PRS are becoming increasingly available through commercial laboratories and in research settings [12].

A risk-stratified screening model of care would offer women PBCRAs followed by risk management programs tailored to their personal risk category. Research in this area shows that population-based BC genetic testing and risk stratification is likely feasible and has the potential to optimize the benefits to harms ratio of NBSPs in a cost-effective manner [13]. The clinical impact of implementing PRS in the familial cancer setting is less well explored.

Effective implementation of risk-stratified screening would require women to accept a PBCRA including a PRS, and then adhere to their risk management plan. As such, women’s engagement with both processes will be major determinants of successful implementation.

To date, the majority of international studies indicate women react positively towards the idea of risk-stratified screening, and that the majority of women would accept a PBCRA combining assessment of lifestyle and genomic risk factors, given that those in the low-risk category continue to be offered screening [14,15,16,17,18]. The potential addition of high-risk gene sequencing to PRS assessment in PBCRA was not addressed, although population screening studies suggest high acceptability [19]. Barriers to risk-stratified screening overlapped with those associated with current NBSPs, namely program access and discomfort associated with mammography. Genomic risk assessment accuracy, cost, and potential side effects have also been reported to influence participation [15]. Although the majority of women report they would accept a PBCRA, some women reported a mistrust that the motivation underlying risk-stratified screening may be economic rather than clinical, and hold concerns over genomic data storage [17,20]. In keeping with these concerns, only the minority of women would accept less or no screening if assessed as low risk [18,21], while others indicated that although they would value a PBCRA, it may not alter their screening behaviour [22].

In the familial cancer clinic context, women with a strong family history of breast cancer but no high- or moderate-risk pathogenic variant were also interested in receiving PBCRA, with 40–60% accepting a PRS assessment in a research setting [23]. Many women integrated their PBCRA as another element in their families’ lived experience of breast cancer. Women who declined demonstrated a higher emotional barrier than those who accepted [23]. Knowledge of PRS altered women’s perception but not necessarily the accuracy of their personal BC risk, without markedly increasing anxiety or distress. There was also little impact on clinical management decision-making [12].

Implementation of personalized BC risk assessment will require a more person-centered approach and a better understanding of how women view this novel model of care for BC early detection and if/how they would wish it to be implemented. Thus, we conducted a qualitative study using focus groups to determine the acceptability of a PBCRA compared to standard current care in three groups of women (general population, women with a strong family history of breast cancer, and women with a *BRCA1* or *BRCA2* pathogenic variant), and to identify what test-specific and test-delivery factors women would take into consideration if offered a PBCRA.

## 2. Materials and Methods

### 2.1. Setting and Sampling

The population of interest was healthy Australian women with and without a family BC history but with no personal history of breast or ovarian cancer. A family history of BC was defined as having at least one first-degree blood relative (parents, siblings, or children) or two second-degree blood relatives (grandparents, aunts/uncles, or nieces/nephews) with a BC diagnosis. Women were recruited through either:

1. LifePool: A prospective cohort study of over 54,000 women recruited after attending *BreastScreen Victoria*: The state breast screening program in Victoria, Australia. Women who previously expressed interest in future research were invited to participate in this qualitative study, or
2. Parkville Family Cancer Center (PFCC): A clinic-based familial cancer service that assesses and manages women at a high familial BC risk.

### 2.2. Recruitment

Women were initially contacted to participate in the study via email. Women that were interested in participating provided consent and were then contacted directly by the study coordinator to arrange attendance at a focus group or personal interview, if preferred. Each participant was identified by a unique study ID and by this means all data were collected and stored in a de-identified but re-identifiable manner.

### 2.3. Focus Groups

Focus groups and interviews were conducted between 13 May 2019 and 3 September 2020 either in person or later on Zoom video conferencing due to COVID-19 restrictions. In order to facilitate an open and comfortable discussion, focus groups were conducted with women who had similar personal histories. The groups comprised:

1. Unaffected Women recruited from LifePool.
2. Unaffected women recruited from PFCC with a strong family history of BC but no familial *BRCA1* or *BRCA2* pathogenic variant identified.
3. Unaffected women known to harbor a pathogenic variant in either the *BRCA1* or *BRCA2* genes.

Focus groups and individual interviews were facilitated by LAK or JW. Before commencing each focus group discussion, all participants completed a short demographic questionnaire.

Each focus group discussion followed a similar format using a guide developed by the authors to facilitate discussion. The focus groups had three elements:

(1) An open discussion of BC risks, risk factors, and risk mitigation or management possibilities,
(2) A short 5-min presentation on genomic-based risk (polygenic and high-risk genes) and potential use of PBCRAs in risk-stratified screening given by a clinical geneticist (AT), and
(3) Discussion of perceptions of the PBCRA, its uses, and potential modes of implementation.

At the end of the focus group with women at population risk or with a strong family history, participants were asked to list the factors and suggest possible alternatives which would impact their decision to have a PBCRA.

### 2.4. Data Collection and Analysis

Focus groups and individual interviews were recorded and transcribed in full by a professional transcription service. Inductive thematic analysis [24] was performed and NVivo12 was used to manage the analysis of data. Each transcript was read and reread by two authors (MS, JW), who developed an initial coding framework. The coding framework and a subset of transcripts were then reviewed by a third author (LK) and further refinements were made to the coding framework. MS and JW then double-coded the data according to the coding framework until a high level of agreement was reached. Given the diversity of the groups, it was difficult to determine if saturation was reached for each of the individual sub-groups [25], however, similar themes were found across all groups, allowing comparison with responses between the different groups.

## 3. Results

### 3.1. Sample Demographics

A total of 31 women participated in the study, with a mean age of 52 years, range 21–70 years. Table 1 presents the characteristics of the study population. Data collection consisted of:

- Two focus groups comprising a total of 15 women with no significant cancer family history recruited from the LifePool cohort study.
- One focus group comprising three women with a strong family history of breast cancer but no pathogenic variant identified through germline genetic testing, recruited from the Parkville Familial Cancer Centre (PFCC).
- Four focus groups and one personal interview comprising 13 women with a strong family history of breast cancer and a *BRCA1* or *BRCA2* pathogenic variant identified, recruited from the PFCC.

### 3.2. Focus Groups and Interviews

Data were coded into three main themes: Overall attitude towards the PBCRA; views on the practicalities of PBCRA implementation, and perceived barriers to PBCRA. These themes and sub-themes are summarized in Table 2.

#### 3.2.1. Theme: Overall Attitude towards the PBCRA

While overall attitudes were positive, there were some caveats to this perception relating to the consequences of a PBCRA. Most participants felt PBCRA results need to be actionable, while others recognized the challenge of changing screening and risk mitigation strategies as risk information alone may not induce behaviour change. The overall impressions can be summarized as follows.

#### The Concept of PBCRA Was Well Accepted, but It’s Use to Inform a Risk-Stratified Breast Screening Program Hinges on Actionability

In each focus group, women responded positively to the concept of a PBCRA. Women understood that multiple factors contribute to risk yet seemed surprised to learn about polygenic risk and its unique inheritance pattern. Due to this individual component of breast cancer risk, participants believed that the PBCRA provides valuable information, especially for women who may develop breast cancer early in life and those with no known family history of breast cancer. Women acknowledged that this information may provide benefits to themselves as well as to other family members. In particular, many indicated a desire for their daughters to be offered the test.

*“You know if I was one of those 25% of [women who develop breast cancer before invited to BreastScreen (Australian NBSP) at age 50] that were offered that service and provided with that information, you know my life could be saved”*.

(68 years, general population)

*“I think the test would facilitate a lot more informed decision-making. I think it wouldn’t be the be-all and end-all, but at least you’d have more information to go off”*.

(45 years, *BRCA1* carrier)

*“It’s sort of like, well this is in your DNA, this is what you’re lumped with, this is your lot in life. And if you had more information about being able to make better informed decisions, for me it comes down to no regrets. So yeah, that… the more information, the better”.*

(42 years, *BRCA2* carrier)

*“Yeah, my daughter’s, yeah her decision making is based very differently to mine and I would kind of, yeah no I would if, I would have it if I thought it was going to impact on her or help her in any way”.*

(67 years, general population)

The majority of women said they would have a PBCRA if offered, without any additional information needed. However, women felt that successful implementation of the PBCRA relies on the availability of appropriate risk management strategies and adequate connection to health and support systems. Furthermore, women found the prospect of the test more appealing when *more* management and screening options were made available to them.

*“If you’ve got enough information that you can make a solid plan then that’s enough information to go ahead”*.

(49 years, general population)

*“You could get more or less screening … but I wouldn’t see any point in taking any kind of genetic testing if you’re not going to do anything about it…”*.

(49 years, general population)

*“Yeah, I think it definitely needs to have a link in terms of the actions that then take place, because it’s just information that might be really scary…”.*

(41 years, *BRCA2* carrier)

#### For Some It Would Not Influence Screening and Risk Mitigation Strategies

Although all women agreed the PBCRA provides valuable information, women, specifically those with a BC family history, find comfort in their current screening practices.

Many women with a BC family history feel that they are at an increased risk of developing BC compared to the general population, regardless of genetic test results, and believe that they benefit from more intensive screening protocols.

*“I feel like knowing the exact percentage doesn’t exactly change the likelihood in my mind. I would still think it’s likely that I might get cancer”*.

(41 years, *BRCA2* carrier)

*“I’d trust it a little bit, but I’d still want to get tested [screened] so I’m doing everything possible to prevent [cancer]”.*

(24 years, strong family history)

*“I privately pay now. I’ve always… I think for me personally would I change the way I act? No. What scares me about that is that it will change the way other people act if it means that they don’t have the same access to screening, to regular screening that they may need.*.

(49 years, strong family history)

#### For Others, It May Augment Decision-Making about Risk Management Strategies

Women who carry a *BRCA1* or *BRCA2* pathogenic variant stated that the information provided by the PBCRA may influence their decisions to undergo prophylactic surgery, specifically the timing of prophylactic bilateral mastectomy and oophorectomy. Women stated they would consider delaying or expediting surgery if their risk was found to be lower or higher than the average *BRCA* pathogenic variant carrier risk, respectively. This was more apparent when considering bilateral oophorectomy because of the side effects of surgically induced early menopause and the consequent impact on fertility.

*“I would think about waiting because of all of the associated risks with early menopause. I’ve already got, I’ve lost bone density already, I know there’s increased heart risks, and they don’t feel good, those risks. So I would have liked to have had that knowledge. So I probably would have waited [for oophorectomy], yeah, and got as close to 50 as I could, I suppose, and then thought about it”.*

(46 years, *BRCA2* carrier)

*“I would be more inclined to hold off with having my ovaries removed than my breasts… I don’t want to go into menopause, that’s what I’m trying to say. So I know I wouldn’t like that to happen very early”.*

(30 years, *BRCA2* carrier)

*“Definitely if I hadn’t had kids there’s no way, I would have gone through it. But I think, yeah, I would consider it or weigh it up more if I knew exactly what the risk was or had a better idea of how much the risk is”*.

(45 years, *BRCA1* carrier)

#### Some Acknowledged the Difficulty of Behaviour Change

Women commented on the autonomy involved in risk mitigation. Many echoed the belief that although this genetic testing and information may be made available to the population, it is up to the individual to act on these results. The participants further emphasized that population-wide genetic testing will not force women to change their lifestyles, behaviors, or cause them to abide by screening recommendations. They likened this to making lifestyle changes for other chronic conditions such as diabetes and heart disease.

*“But on the same token, it has to be free will. I mean, people are still allowed to smoke and that’s their choice. I mean, my auntie’s BRCA2 [gene positive] and she is in her 50s. She’s not getting her breasts removed, she’s just going to be happy with the yearly MRIs. You know, that’s up to her, we all have like, you know, your different paths. It’s like a choose your own adventure book”.*

(42 years, *BRCA2* carrier)

*“Yeah, but I mean do people change their behaviors when they’ve been told oh, you’re at a high risk of Diabetes [type] 2? Do you see people changing their lifestyle you know to reduce that risk?”*.

(58 years, general population)

*“Like as much as I think it’s a pretty good idea, most people in the population know that it’s not a good idea to be overweight. And I’ve just lost ten kilos because I got confronted by seeing a heart specialist, and it made me do something. But it wasn’t, like I’m sixty—it took until I was 65 to actually confront the issue that I needed to do something about my longevity. Now some people are going to be, take that more seriously”*.

(65 years, general population)

### 3.2.2. Theme: Women Addressed Potential Deterrents to Genome-Based Personal Risk Assessment

All women agreed that the PBCRA has the potential to cause anxiety or stress, especially when identifying women at above-average BC risk. The additional knowledge may affect an individual’s peace of mind or create feelings of panic, helplessness, and the perception that cancer is inevitable. Some women feel it may be better not to know their cancer risk, however, this is dependent on their personality as some individuals are more prone to anxiety.

*“Just the anxiety if you know, of living with that. Some people get very stressed like me (laughs)”*.

(58 years, general population)

*“Like I think it could be powerful, but I almost like put it like some kind of crazy pandemic of everyone just thinking they’re going to die of breast cancer”.*

(36 years, BRCA1 carrier)

*“Just in terms of the general population, I guess having the test can be, can cause a bit of anxiety, I think. So is it worth every single person in the population going through that, and a potential period, of six weeks of feeling quite anxious, about something that may not mean that much, if their results are low or whatever it is. That obviously would be reassuring, but I’m sure that there would still be, and depending on what emotional state they’re in at the time that they get that I think that should be looked at, because I think it can be anxiety inducing and that’s probably a consideration as well”.*

(41 years, *BRCA2* carrier)

Some participants discussed the potential for genetic discrimination. Women were worried that an increased risk may be viewed as a pre-existing condition and thus could impact insurance policies, superannuation, and employment opportunities.

*“Oh yeah well I have concerns about that sort of knowledge being put out there for them to sort of… insurance, superannuation, and those sort of formal things that you wouldn’t want people to be discriminated against because of their genetic makeup”.*

(68 years, general population)

### 3.2.3. Theme: Views on the Practicalities of PBCRA Implementation

Participants were asked about the practicalities of implementing the PBCRA, and the main themes that emerged described the importance of trusted organizations offering and endorsing the test, ease of testing, and appropriately sensitive and supportive return of results. There was no clear consensus on the age at which women would most benefit from the test.

#### Trust in Testing Procedures

All women preferred a test endorsed and performed by people or institutions they were familiar and comfortable with, such as their physicians, the government, and *BreastScreen Australia*. Women report they want to feel safe and have access to appropriate resources if needed, and want to receive consistent advice from all their healthcare providers.

*“Oh well I’d, my preference would be at the BreastScreen that they test you there and then you go, if you need to go back for your results there. They’ve got the counsellors or presumably got the counsellors in place and the sources to send you off to or advise you on what the next procedure is I presume, because they are specializing in breast cancer”.*

(58 years, general population)

*“You can give us this testing but if I go back to my breast specialist and they’re not on the same page, I’m going to get conflicting discussions again, which I’ll end up just probably going with my breast specialist, the person in front of me. So you really need to bring the specialists onboard with these, this testing and the power of the testing”.* (46 years, *BRCA2* carrier)

#### Ability to Connect with Target Age Group

A topic of debate was the age at which the test should be offered to women. Issues regarding consent, mental maturity, responsibility, and the value of genetic information were discussed.

*“I mean at what age do we kind of say okay this kind of person is definitely in a position to be responsible about the information they’d been given”*.

(67 years, general population)

*“Like, so life, life, every aspect of your life has an impact on your decision-making processes. So it all depends on where you are and what stage and how you’re feeling about things because they will all impact”.*

(50 years, *BRCA2* carrier)

Participants proposed three target populations:

**Teenagers:** Some women believed that testing should be introduced along with the human papilloma virus vaccination in school systems. They perceived that teenagers may not find value in genetic testing, but it may be appropriate to educate this age group about genetic BC risk alongside other health messages (such as smoking).

*“If they choose to ignore it, that’s fine they probably will you know at 14 or something. But at some stage it’ll be like oh ding. I shouldn’t be binge drinking or I shouldn’t be whatever they’re up to. Or I better go and get these results”*.

(58 years, general population)

**Young adults***(20–30 years):* The majority of women in this study felt that testing should target young adults. This would capture high-risk women who develop breast cancer before age 50, while accounting for maturity and minimizing unnecessary angst.

*“You really want to be hitting adulthood as a person to be able to handle maybe some news that’s going to impact on how you manage your healthy for the rest of your life. Even if you never get sick, you’re going to have to manage it. So, I think you probably want to be well and truly in that adult stage”.*

(63 years, general population)

**BreastScreen***(50 years):* Some women thought the test should be introduced when women begin *BreastScreen* since women already expect to begin screening at age 50 years, for both breast and bowel cancers.

*“I guess most people who are high risk it’s going to be, they’re going to know they’ve had an aunt or a mother or sister…You could request it earlier if you’ve got a family history”*.

(49 years, general population)

#### Ease of Testing

Participants, particularly those at general population risk, stated that people are more likely to participate if the PBCRA is convenient to access. Factors influencing convenience included: cost, location, effort, type of test (blood vs. saliva, separate test vs. added to standard blood work), and appointment type.

*“I think the easier you make it you could get to, in any way that you make it easy to do. Easy to access, easy to send off, cheaper, you know all of those things are going to get more people involved”*.

(49 years, general population)

*“I have a lot of blood tests for other conditions and if the doctor said that to me and it was just, I’ll tick this box, I probably actually would have”*.

(68 years, general population)

#### Return of Results

Compared with women at population risk, women with a family history who had undertaken genetic testing seemed less concerned with how convenient the PBCRA was to undertake. These women focused their discussion more on how PBCRA results should be returned. Factors they discussed included: choosing how to receive results (phone call, letter, in person), time and space to process information, providing information in a way that is easily digestible/understood, and the use of graphics and visualizations.

*“I think if you’re given that information in a pamphlet, leaflet, all of that sort of stuff where you can actually read the pros and cons, the possibilities, without going into graphic detail, but then you can sort of take it away in your own time and then, you know, in four weeks’ time you organise another meeting with those people. I think that would be a really good way of delivering information because otherwise you’re chasing it and you’re in shock, and you don’t really know who to talk to or what to ask or how to say it”.*

(48 years, *BRCA1* carrier)

*“I personally think I would have liked the whole experience to be quite different of being told… like I, I don’t like getting, well, you know, crying in front of others. And so I would have much preferred to have received a letter or a phone call and then a few days later been able to go in in a calm sort of way, process the information and then go back a week later and meet again and hear it for a second time or hear more information”.*

(30 years, *BRCA2* carrier)

*“I was going to say that the graphs are not difficult to get. If you just take your age at that point and move onwards from there, say up to 80%, and just draw one line with BRCA and the other one with your other risk factors, I think it would be pretty straightforward and most women would get that”.*

(30 years, *BRCA2* carrier)

#### Effect of Implementation of PBCRA on Population Breast Cancer Screening

Women discussed that risk stratification could be used to modify current breast cancer screening methods. Proposed changes included earlier and/or more frequent breast screening for those at high risk, and decreased or delayed screening for those at lower risk. Compared to current mammography screening methods, women believed that a risk-stratified screening program will be cheaper and more efficient.

*“You get into the mammogram program at 25 instead of waiting until you’re 50 for example”*.

(49 years, general population)

*“I think it’s, for me if I was low risk then I probably wouldn’t have, I probably wouldn’t have the mammogram or as often”.*

(67 years, general population)

*“If you’re at a higher risk then you are scheduled for more screening on a more regular basis”*.

(58 years, general population)

However, some women stated that it may be difficult for the population to agree to new screening standards, as they have a sense of security with their current screening practice. They felt particularly concerned for women deemed low risk, regardless of their family histories, who may be offered less screening compared to the average risk women.

*“It’s hard to accept. It’s like with the pap smear. My doctor has said I only need to have one every five years now, but I don’t know. I think I would rather have whatever I was having them before every two years just for that peace of mind”.*

(48 years, *BRCA1* carrier)

*“I wonder if it’s generational though because I look at you know my generation and with things like the bowel and the mammogram, you’re just in this system that you’re expecting things to happen you know in a particular way. So, it offers comfort that you’ve got these regular points where the government pretty much intervenes and says we’re going to look after your health”.*

(63 years, general population)

## 4. Discussion

The results from this study are novel as we have been able to compare and contrast the views of women across the BC risk continuum from women with previously identified *BRCA1* and *BRCA2* pathogenic variants in the FCC setting, to those participating in the Australian NBSP, *BreastScreen Australia,* recruited through LifePool. These contextual considerations are critical, because any implementation of PBCRA is likely to be in one or both of these settings, and will affect women differently depending on their age, risk level, and lived experience of cancer. Women recruited from the FCC bring with them the experience of previous genetic testing, and how it is currently implemented, while those recruited from Lifepool provide the perspectives of women with no experience of genetic testing.

In keeping with other studies [18,26], participants responded positively to the idea of a genetic test- based personal risk assessment, while raising a number of issues that will need to be key considerations in any future implementation design.

Actionability of test results was highly valued, and this meant different things for different risk groups. For those at population risk, adjusting the age of commencing screening or the interval of screening was considered valuable. However as has been previously reported, the ability to identify women at higher risk in need of more intense surveillance was valued more highly than the ability to reduce screening in lower-risk women, which may be a barrier to implementation [18]. In keeping with this, women at population risk or with a strong family history indicated that high-risk gene sequencing or other disease risk profiles for treatable conditions could be integrated into the genetic test. In contrast, women carrying a *BRCA1* or *BRCA2* pathogenic variant would consider delaying oophorectomy if found to have low ovarian cancer PRS assessment, as delaying the onset of surgically induced menopause was appealing.

Most participants noted that following a PBCRA, advice from health professionals regarding altered breast screening and risk-reducing interventions are more likely to be followed than advice to change risk behaviors such as reducing either alcohol consumption or weight, or stopping smoking. A major caveat to this was that advice to reduce screening in individuals whose personal risk indicates a low or average risk would be less likely to change screening behaviour if they perceived themselves to be at increased risk due to their lived experience of cancer in the family, a concept previously described [20]. This did not appear to relate to concerns over test accuracy. However, this sample of women may be more trusting in the test accuracy and healthcare system than most, as they have either undertaken a genetic test through the PFCC or have participated in *Breastscreen Australia*. The women reported that current screening programs provide a sense of security, and highlighted the importance of providing PBCRAs through a trusted institution which they believed would ensure test accuracy.

The risk of increased anxiety in tested populations, uncertainty when waiting for results, and concerns for genetic discrimination are all social issues that will need to be addressed when implementing PBCRAs in the future. It is possible these concerns may diminish over time if having a PBCRA is normalized within society.

There was less consensus on the practical details of PBCRA implementation. Participants did not agree on an ideal age to offer testing, with suggestions ranging from 15 to 50 years of age; 50 years being the age at which women are invited to NBSP in Australia. Women often discussed their daughters when asked about an ideal age, again highlighting that women value the applicability of genetic information towards their children [27]

Similarly, ease of access to testing was less important for those with a family history than for those without. Participants recruited from Lifepool (originally recruited through *BreastScreen Victoria*) were happy for the test to be offered through *BreastScreen* (taking into account the above caveats). However, those who had undergone genetic testing in the FCC were concerned with how women would receive different risk profiles and management messages from test providers and their clinical specialists. It will be important to ensure health professionals interpret and make similar management recommendations based on PBCRA results.

The views of women with a strong family history who had undergone genetic testing should be viewed in light of their lived family experience. These women were less concerned with barriers to the PBCRA, focusing their discussion more on how results should be returned rather than how convenient the test should be to access. These results may be interpreted as suggesting such women are more likely than those with no family history to participate in PBCRA and subsequent risk-stratified screening because of their experience of seeing family members affected by breast cancer. However, these women had already experienced genetic testing, so it is possible that the women sampled were less concerned with the logistics of accessing the test as they have already demonstrated they have the means to overcome any barriers to genetic testing. Consequently, further study is required to determine if these views are shared by women with a strong BC family history who have not attended an FCC.

Most of the proposed implementation processes collected at the end of the focus group discussions with women at population risk or with a strong family history arose during the preceding discussions. The targeted discussion in which they were explicitly collected was not included in the thematic analysis. They suggest that women value ease of access over specialist involvement, and more information over a more limited approach, with the caveat that the test was provided by a socially accepted, “trusted” source. These criteria were explored further using a multicriteria decision analysis approach in a larger group of women to better understand the relative impact on PBCRA participation rates, should such approaches be adopted (Wheeler et al., manuscript accepted, Genetics in Medicine).

## 5. Recommendations

Moving towards a population-based risk stratified breast screening program in Australia comes with challenges. This study demonstrates that the implementation of a PBCRA is acceptable, however women are particularly concerned with the actionability of results and the uncertainty that comes with less screening, especially for those who view themselves as high risk. In order to mitigate the anxiety that women feel, it will be important to educate the wider population, both women and healthcare providers, on breast cancer genetics, risk factors, and determine optimal risk-category-specific screening methods. Additionally, in order to make the results more tangible, information obtained through the PBCRA should be tailored for each specific risk category (i.e., low, medium, and high risk), and explicitly acknowledge and address the role that the lived experience of cancer may have on a woman’s risk perception. Systems must be in place to appropriately deliver genomic information with adequate emotional and psychological support where needed.

Further research must be done regarding the execution of the test. This includes: method of delivery, the age at which the PBCRA should be offered, and the return of the results. Many factors impact these decisions and information gathered from a diverse sample of women would allow for a smooth transition from the current model. Our study highlights how women in Australia feel a sense of security with current practices, and it is important to maintain this trust when planning any changes in the future. Although the study was conducted in Australia, the concept of risk stratified breast cancer screening is internationally applicable, especially in countries with well-established population-based screening programs.

## 6. Study Limitations

The “general population” woman recruited for this study were sampled from a cohort of women (LifePool) who expressed interest in future research through BreastScreen and as such may be more biased towards early adoption. These women may be more experienced, trusting, and accepting of current and future breast cancer screening methods compared to others as they had previously undertaken mammography through BreastScreen. Additionally, the participants in our focus groups were not racially, linguistically, or socioeconomically diverse and women with a family history who have the potential to benefit most were under-represented. Thus, our sample is relatively homogeneous, and we appreciate this as a limitation of the study and its generalizability to a wider population of Australian women. Future work could be done to explore a more diverse group of women’s interests in the PBCRA and population-based breast screening methods.

## Figures and Tables

**Table 1:** Demographic characteristics of participants.

Characteristics	
Age: 20–29 30–39 40–49 50–59 60–69 70+	(*n* = 31) 2 3 9 6 10 1
Recruitment: LifePool PFCC	(*n* = 31) 15 16
Genetic Test Results of PFCC Participants: No pathogenic variant identified *BRCA1* pathogenic variant identified *BRCA2* pathogenic variant identified	(*n* = 16) 3 5 8
Highest Education Status Achieved: University or higher degree High school certificate or trade/apprenticeship No school certificate or other qualifications Unknown—demographic information not returned	13 4 7 7

**Table 2:** Summary of themes and subthemes.

**Themes**	**Subthemes**
Overall attitude towards the PBCRA	The PBCRA was well accepted, but it’s use to inform a risk-stratified breast screening program hinges on actionability
	For some it would not influence screening and risk mitigation strategies
	For others, it may augment decision-making about risk management strategies
Perceived barriers to PBCRA	Anxiety and stress are inevitable for some Concerns of genetic discrimination
Views on the practicalities of PBCRA implementation	Trust in testing procedures
	Ability to connect with target age group
	Ease of testing
	Return of results

## Data Availability

De-identified data collected and analyzed in this study are available from the corresponding author.
